# Hiatal herniation of the stomach and pancreas in a patient with oxygen desaturations

**DOI:** 10.3402/ljm.v8i0.23288

**Published:** 2013-12-04

**Authors:** Daniele Torres, Gaspare Parrinello, Mauro Cardillo, Michele Bellanca, Giuseppe Licata

**Affiliations:** Dipartimento Biomedico di Medicina Interna e Specialistica, Azienda Ospedaliera Universitaria Policlinico ‘Paolo Giaccone’, Università degli Studi di Palermo, Palermo, Italy

**Fig. 1:**
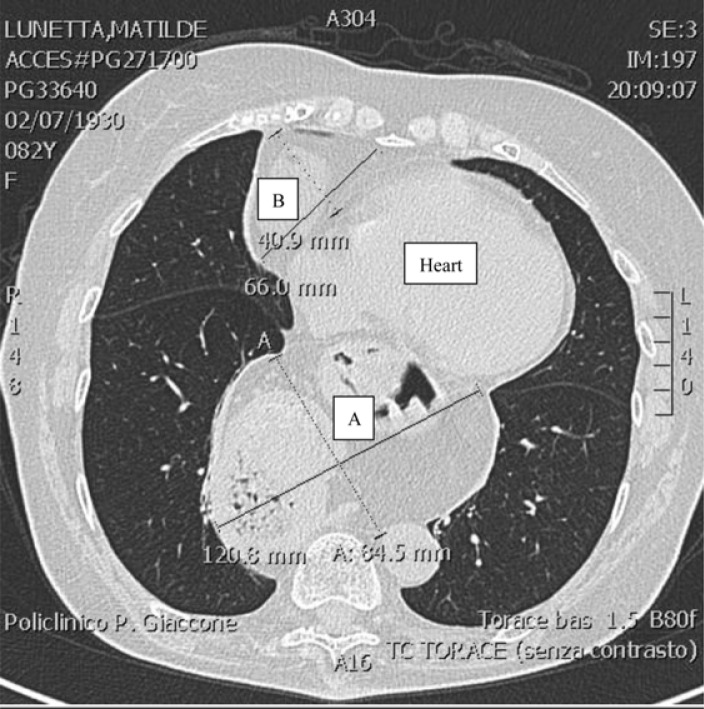
Chest computed tomography demonstrating the complete hiatal hernia compressing right atrium and ventricle with dimensions (A). In the lower retrosternal right side, a diaphragmatic hernia with fluid and adipose content is also present (B).

Hiatal hernia (HH), a neglected cause of cardio-respiratory symptoms, is a frequent entity characterized by the displacement of the gastro-esophageal junction and part of the stomach into the mediastinum. Although often asymptomatic, HH may also exert a wide spectrum of clinical presentations due to cardio-pulmonary compression, including acute cardio-vascular events such as arrhythmias, post-prandial syncope, angina-like chest pain, recurrent acute heart failure, hemodynamic collapse, electrocardiographic changes (T-wave inversion, ST elevation) simulating myocardial ischemia or pericarditis, and respiratory manifestations that can range from exercise intolerance and dyspnea on exertion to alteration of pulmonary function tests. Further cardiac complications include the formation of gastro-pericardial fistula and subsequent pericardial effusion (1–5). An 82-year-old woman complained of 1-week symptomatology characterized by frequent episodes of dyspnea on mild exertion associated with respiratory desaturation measured at pulse oximeter (SpO2 85%). The Doppler echocardiogram, performed outside the hospital, showed the worsening of the already known pulmonary hypertension (60 mmHg vs. 48 mmHg measured 11 months before). The patient was hospitalized at the Internal Medicine Department to investigate this clinical condition. On admission, she was alert, normotensive and eupneic at rest, and afebrile. Her medical history included: essential arterial hypertension, stage III chronic kidney disease (eGFR 40 ml/min), mild to moderate restrictive broncho-obstructive dysventilatory syndrome associated with kyphoscoliosis, and gastro-esophageal reflux disease. The patient denied any recent history of angina, palpitations, fever, and cough. No signs/symptoms of heart failure were observed. The 12-lead electrocardiogram (ECG) demonstrated sinus rhythm (55 bp/min) with bifascicular block (right bundle block and left-anterior hemiblock). Laboratory tests, including blood gas analysis, cardiac troponin, complete blood count, C-reactive protein and erythrocyte sedimentation rate, were within normal reference values. A two-dimensional transthoracic Doppler echocardiogram, using all standard and modified apical and parasternal views, was rapidly performed. It revealed normal volume of all cardiac chambers, concentric remodeling of left ventricle with preserved ejection fraction, altered left ventricular relaxation, mild to moderate tricuspid insufficiency, mild increase of the estimated systolic pulmonary artery pressure (40 mm Hg) and normal estimated left ventricular filling pressure (*E*/*E* ^1^=8.5 mmHg). However, an unusual echolucent mass compressing the right atrium was found. The first suspect was an HH that was confirmed by esophagogastroduodenoscopy. Chest computed tomography (Fig. 1) showed into the mediastinum, another structure, with dimensions as a second heart, which is determined by the complete herniation of the stomach and pancreatic body and tail. In the lower retrosternal right side, a diaphragmatic hernia with fluid and adipose content was also found. Neither structural nor inflammatory lung diseases were observed. The patient was referred to a surgical consultation to further assess the repair of HH. The surgeon raised the suggestion of surgery, but the patient refused. After 11 months, the patient was alive.

This report illustrates an uncommon case of giant HH with complete herniation of the stomach and a great part of the pancreas. In this case, the massive HH was a precipitating factor of arterial pulmonary hypertension and dysventilatory syndrome with respiratory desaturation likely due to its compressive effects on the lungs and heart. Massive HH may be considered in differential diagnosis of progressive development of dyspnea.

*Daniele Torres* Dipartimento Biomedico di Medicina Interna e Specialistica Azienda Ospedaliera Universitaria Policlinico ‘Paolo Giaccone’ Università degli Studi di Palermo Palermo, Italy E-mail: daniele_torress@libero.it *Gaspare Parrinello* Dipartimento Biomedico di Medicina Interna e Specialistica Azienda Ospedaliera Universitaria Policlinico ‘Paolo Giaccone’ Università degli Studi di Palermo Palermo, Italy *Mauro Cardillo* Dipartimento Biomedico di Medicina Interna e Specialistica Azienda Ospedaliera Universitaria Policlinico ‘Paolo Giaccone’ Università degli Studi di Palermo Palermo, Italy *Michele Bellanca* Dipartimento Biomedico di Medicina Interna e Specialistica Azienda Ospedaliera Universitaria Policlinico ‘Paolo Giaccone’ Università degli Studi di Palermo Palermo, Italy *Giuseppe Licata* Dipartimento Biomedico di Medicina Interna e Specialistica Azienda Ospedaliera Universitaria Policlinico ‘Paolo Giaccone’ Università degli Studi di Palermo Palermo, Italy
